# Unveiling the Rational Development of Stimuli-Responsive Silk Fibroin-Based Ionogel Formulations

**DOI:** 10.1021/acs.chemmater.3c00303

**Published:** 2023-07-20

**Authors:** Talia A. Shmool, Laura K. Martin, Andreas Jirkas, Richard P. Matthews, Anna P. Constantinou, Devkee M. Vadukul, Theoni K. Georgiou, Francesco A. Aprile, Jason P. Hallett

**Affiliations:** † Department of Chemical Engineering, Imperial College London, South Kensington Campus, London SW7 2AZ, U.K.; ‡ Department of Engineering Science, University of Oxford, Parks Road, Oxford OX1 3PJ, U.K.; § Department of Bioscience, School of Health, Sports and Bioscience, University of East London, Stratford, London E15 4LZ, U.K.; ∥ Department of Materials, Imperial College London, South Kensington Campus, London SW7 2AZ, U.K.; ⊥ Department of Chemistry, Molecular Sciences Research Hub, Imperial College London, London W12 0BZ, U.K.; # Institute of Chemical Biology, Molecular Sciences Research Hub, Imperial College London, London W12 0BZ, U.K.

## Abstract

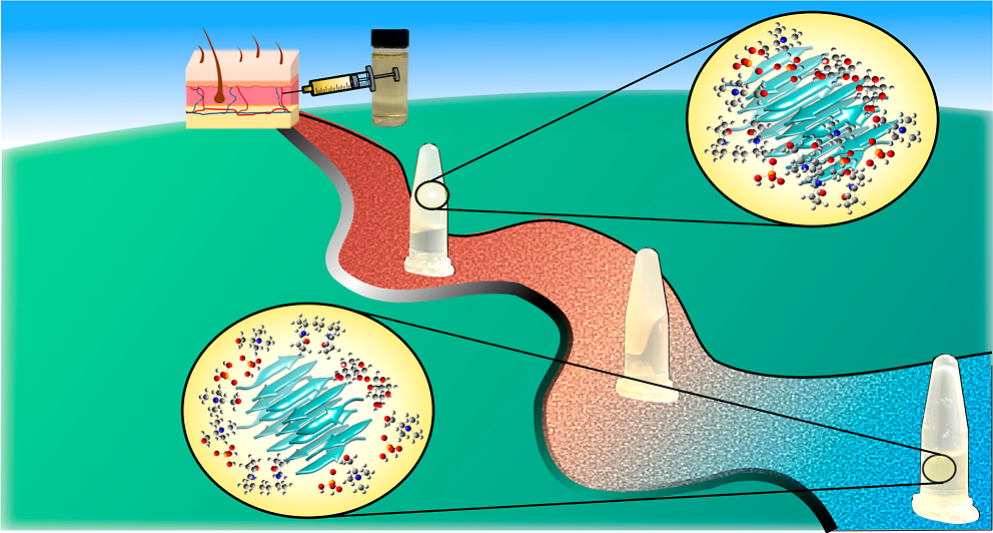

We present an approach for the rational development of stimuli-responsive ionogels which can be formulated for precise control of multiple unique ionogel features and fill niche pharmaceutical applications. Ionogels are captivating materials, exhibiting self-healing characteristics, tunable mechanical and structural properties, high thermal stability, and electroconductivity. However, the majority of ionogels developed require complex chemistry, exhibit high viscosity, poor biocompatibility, and low biodegradability. In our work, we overcome these limitations. We employ a facile production process and strategically integrate silk fibroin, the biocompatible ionic liquids (ILs) choline acetate ([Cho][OAc]), choline dihydrogen phosphate ([Cho][DHP]), and choline chloride ([Cho][Cl]), traditional pharmaceutical excipients, and the model antiepileptic drug phenobarbital. In the absence of ILs, we failed to observe gel formation; yet in the presence of ILs, thermoresponsive ionogels formed. Systems were assessed via visual tests, transmission electron microscopy, confocal reflection microscopy, dynamic light scattering, zeta potential and rheology measurements. We formed diverse ionogels of strengths ranging between 18 and 642 Pa. Under 25 °C storage, formulations containing polyvinylpyrrolidone (PVP) showed an ionogel formation period ranging over 14 days, increasing in the order of [Cho][DHP], [Cho][OAc], and [Cho][Cl]. Formulations lacking PVP showed an ionogel formation period ranging over 32 days, increasing in the order of [Cho][OAc], [Cho][DHP] and [Cho][Cl]. By heating from 25 to 60 °C, immediately following preparation, thermoresponsive ionogels formed below 41 °C in the absence of PVP. Based on our experimental results and density functional theory calculations, we attribute ionogel formation to macromolecular crowding and confinement effects, further enhanced upon PVP inclusion. Holistically, applying our rational development strategy enables the production of ionogels of tunable physicochemical and rheological properties, enhanced drug solubility, and structural and energetic stability. We believe our rational development approach will advance the design of biomaterials and smart platforms for diverse drug delivery applications.

## Introduction

A major hurdle in drug development is designing and engineering controlled drug delivery systems.^1,2^ Such systems exhibit capabilities to maximize the efficacy of a given active pharmaceutical ingredient (API), prolong retention at the desired target site, and reduce the toxicity and dose required.^1,2^ In this regard, stimuli-responsive systems can offer spatial-, temporal-, and dosage-controlled drug delivery.^1−3^ Stimuli-responsive systems exhibit sensitivity to a specific applied stimulus, such as temperature changes, prompting spatiotemporally controlled release of the desired drug.^3^ Particularly attractive are thermoresponsive systems, effective platforms for local, controlled, and sustained drug delivery.^4,5^ However, the majority of stimuli-responsive systems require complex chemistry, compromising biocompatibility and scale-up.^3−5^ Furthermore, thermoresponsive carriers should optimally retain the desired drug at 37 °C, an additional challenge for developing effective local and controlled drug delivery systems.^4,5^ We are motivated to overcome these impeding obstacles and explore unconventional avenues in order to advance the development of intelligent drug delivery systems.

Ionogels are intriguing hybrid materials exhibiting high transparency, electroconductivity, thermal and structural stability.^6−8^ Conventionally, an ionogel is comprised of a given ionic liquid (IL), consisting of a cation and an anion, entrapped within a polymer network. The majority of studies examining ionogels for biomedical applications resort to imidazolium-based ILs, showing high conductivity and self-healing characteristics.^9^ However, in general, imidazolium IL-based ionogels are of high viscosity, poor biocompatibility, and low biodegradability; limiting the exploitation of existing ionogels in pharmaceutical applications.^10^ Given the highly tunable mechanical and structural properties of ionogels,^6−9,11,12^ we were inspired to leverage rational formulation design to develop diverse, proof-of-concept, thermoresponsive ionogel formulations. These will encompass ionogels which form at distinct periods under 25 °C storage and instantaneously upon heating from 25 to 60 °C, optimally below 37 °C.

In our work, an ionogel formulation is considered a viscoelastic material comprising an IL confined within a polymer matrix and incorporated traditional pharmaceutical excipients serve as macromolecular crowding agents. To rationally design ionogel formulations, it is key to modulate properties such as composition, size, surface charge, stability, responsivity, and strength. We select choline dihydrogen phosphate ([Cho][DHP]), choline acetate ([Cho][OAc]), and choline chloride ([Cho][Cl]) as our model ILs. Previously, these ILs have been shown to lower formulation viscosity, increase gel mechanical strength, induce gelation, and modulate the gelation temperature of diverse gel systems.^13−18^ These ILs have been demonstrated as biocompatible and shown to enhance API solubility and suppress protein aggregation.^13,14,19−22^ Our choice of the silk fibroin biopolymer was premised in that it is biocompatible, biodegradable, and bioresorbable and offers tunable mechanical properties.^23,24^ However, employing facile methods to prepare silk fibroin-based materials can result in aggregate formation, reduced mechanical strength, insufficient response to environmental stimuli, and limit fabrication to soft tissue medical materials.^23−25^ Thus, although numerous studies have explored silk fibroin-based platforms for drug delivery applications,^23−30^ further research is required. Given that low drug solubility is a major issue in formulation development, we were motivated to study the long-acting barbiturate phenobarbital as our model drug, as it would benefit from enhanced solubility.^1,2,31^ We include proportions of our biocompatible ILs and silk fibroin biopolymer previously found optimal for directing gel self-assembly, exhibiting stimuli-responsive properties, and increasing the mechanical strength and the structural and thermal stability of diverse ionogels and polymer-based platforms.^6,32−34^ We limit the water content and create formulation buffers comprising tween 20, histidine, trehalose, and sucrose in the absence and presence of glycerol. These traditional pharmaceutical excipients were included at proportions shown to enhance API solubility, extend shelf-life, promote the self-assembly of stimuli-responsive gels, and delay gelation processes.^13,35−40^ We also create formulations including polyvinylpyrrolidone (PVP) at proportions shown to promote stimuli-induced gelation, enable controlled drug release, and improve the mechanical strength of imidazolium IL-based ionogels.^26,32,41,42^ Notably, by employing a facile production process, eliminating the requirement of complex chemistry, ultimately we aim to facilitate the implementation and translatability of our approach for ionogel formulation development into the pharmaceutical sector. To our knowledge, our rational design and systematic approach involving synergistic combinations of these excipients and choline-based ILs to develop stimuli-responsive silk fibroin-based ionogel formulations for niche pharmaceutical applications has yet to be explored.

We visually determine phenobarbital solubility in each formulation and the period required for ionogel formation under 25 °C storage. We conduct dynamic light scattering (DLS) and zeta potential measurements to determine the aggregation propensity and surface charge of each system. We heat the formulations from 25 to 60 °C immediately following preparation to evaluate the thermoresponsive properties of each system. We conduct rheology experiments to confirm ionogel formation and determine ionogel strength. We perform transmission electron microscopy (TEM) and confocal reflection microscopy (CRM) to examine the morphology of the ionogel networks. Notably, the energetic driving forces of hydrogen bonding in multicomponent IL systems have long been a subject of investigation, yet to be resolved. Thus, we were motivated to perform density functional theory (DFT) calculations to determine the structural and energetic properties and probe the nature of hydrogen bonding of the diverse ionogel formulations.

## Results

### Phenobarbital Solubility in Ionogel Formulations

**Figure 1:**
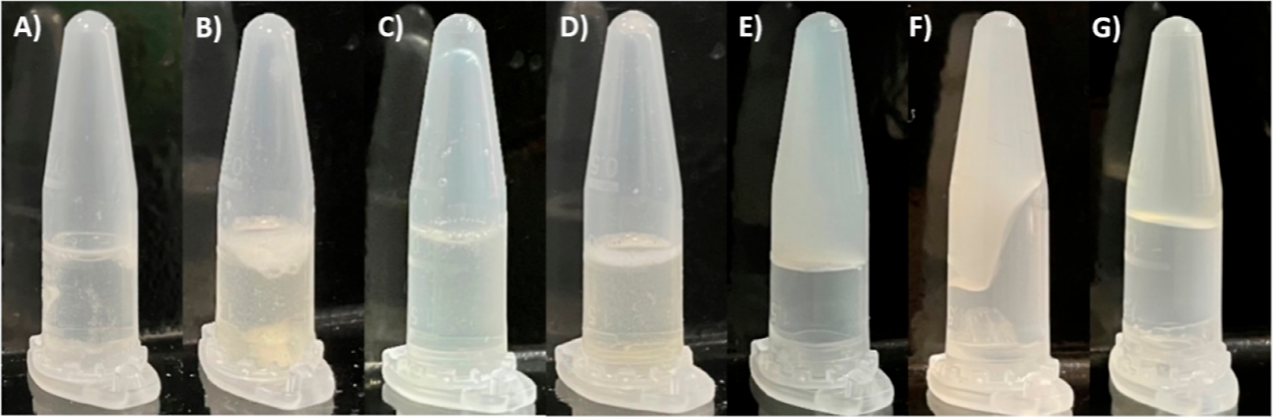
Photographs of phenobarbital in (A) F3 and (B) PVP-F3 lacking IL, (C) fresh F3[Cho][OAc] and (D) fresh PVP-F3[Cho][Cl], (E) F3[Cho][OAc]-ionogel following 1 day, and PVP-F3[Cho][Cl] following (F) 7 and (G) 12 days under 25 °C storage. The yellow tinge in B,D,F, and G is due to the nature of the PVP ingredient.

Phenobarbital is poorly soluble in aqueous solutions, despite the inclusion of traditional solubility enhancing excipients.^31,44^ In agreement, we found that phenobarbital was largely insoluble in formulations lacking IL (Figures 1A,B and S1A). However, in the presence of IL phenobarbital solubility was significantly enhanced, showing instantaneous solubility in [Cho][OAc] and [Cho][DHP] containing formulations, relatively lower for formulations containing [Cho][Cl] (Figures 1C,D and S1B). Specifically, formulations containing [Cho][Cl] and lacking glycerol required, on average, 6 h to show complete solubility. Our findings that phenobarbital solubility was promoted in the presence of IL and glycerol is also consistent with previous work.^45^

### Ionogel Formation Period under Ambient Temperature Storage

Following preparation, our formulations were stored under 25 °C, and over the course of two months we failed to observe gel formation in formulations lacking IL. In contrast, we observed ionogel formation for each IL containing formulation stored under 25 °C (Figures 1E–G and S1C). In the absence of PVP we found that [Cho][OAc] containing formulations required the shortest period for ionogel formation, followed by [Cho][DHP] and longest period observed for [Cho][Cl] (Table 1). In the presence of PVP, [Cho][DHP] containing formulations showed the least delayed period for ionogel formation, followed by [Cho][OAc] and longest period found for [Cho][Cl] containing formulations (Table 1). Overall, formulations containing trehalose and glycerol required the shortest period for ionogel formation, further reduced in the presence of PVP. For example, F3[Cho][OAc] and F4[Cho][OAc] containing glycerol showed ionogel formation following 1 day compared to a 7 day formation period required for the F1[Cho][OAc]-ionogel lacking glycerol. As well, the formation period of the PVP-F1[Cho][Cl]-ionogel was 14 days compared to 32 days for the F1[Cho][Cl]-ionogel. Notably, we examined the ionogel formulations stored under 25 °C over the course of two months and found each maintained structural integrity over the observation period.

**Table 1:** Ionogel Formation Period under 25 °C Storage

ionogel formulation	ionogel formation period (days)
F1[Cho][OAc]	7
F1[Cho][DHP]	14
F1[Cho][Cl]	32
F2[Cho][OAc]	4
F2[Cho][DHP]	10
F2[Cho][Cl]	24
F3[Cho][OAc]	1
F3[Cho][DHP]	4
F3[Cho][Cl]	18
F4[Cho][OAc]	1
F4[Cho][DHP]	7
F4[Cho][Cl]	23
PVP-F1[Cho][OAc]	12
PVP-F1[Cho][DHP]	7
PVP-F1[Cho][Cl]	14
PVP-F2[Cho][OAc]	10
PVP-F2[Cho][DHP]	6
PVP-F2[Cho][Cl]	14
PVP-F3[Cho][OAc]	7
PVP-F3[Cho][DHP]	3
PVP-F3[Cho][Cl]	12
PVP-F4[Cho][OAc]	7
PVP-F4[Cho][DHP]	1
PVP-F4[Cho][Cl]	12

### Aggregation Propensity and Surface Charge of Developed Formulations

**Figure 2:**
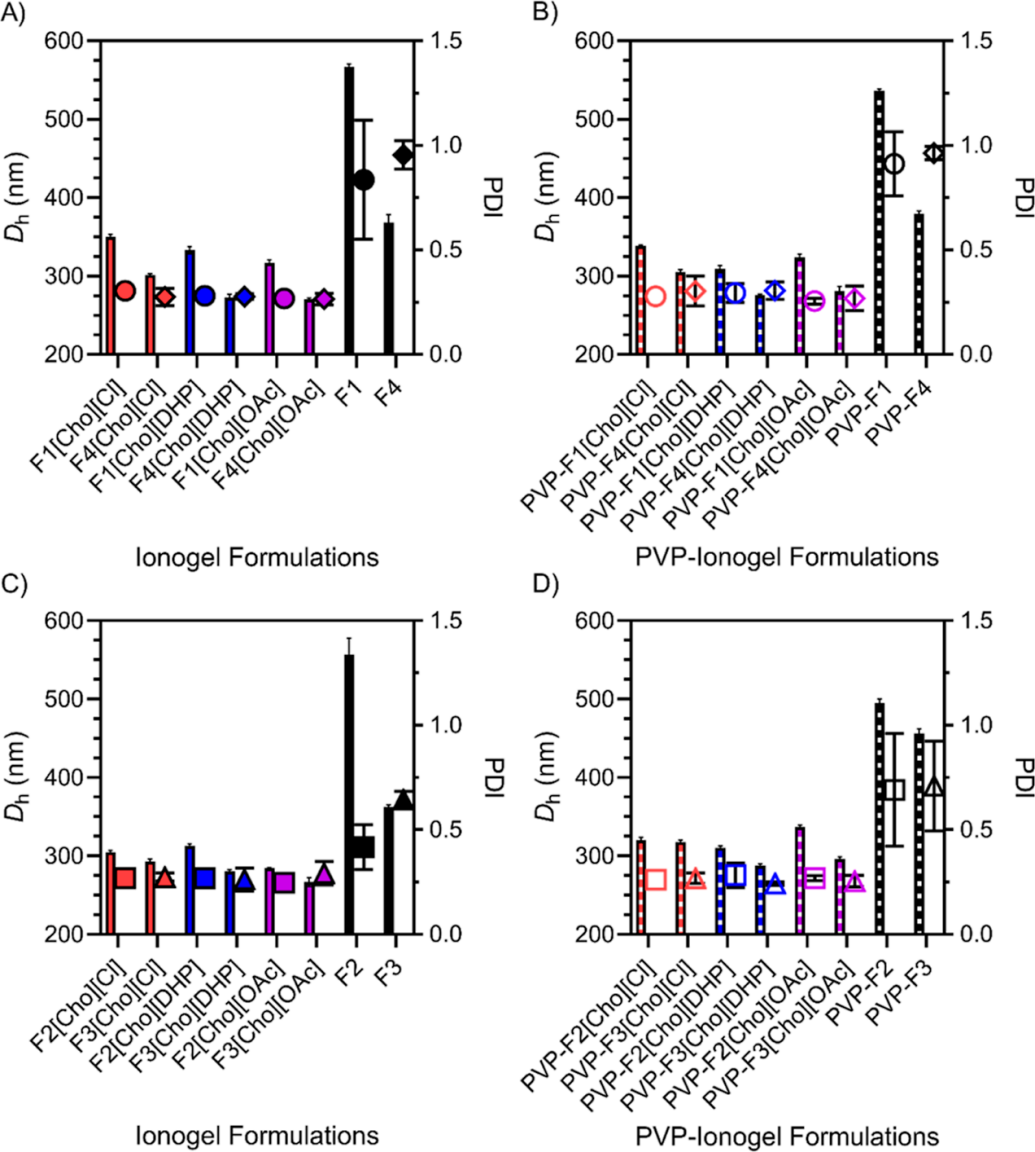
Hydrodynamic diameter (*D*_h_, bars) and polydispersity index (PDI, symbols) values for [Cho][Cl], [Cho][DHP], and [Cho][OAc]-ionogel formulations represented in red, blue, and purple, respectively. Formulations lacking IL are shown in black. F1, F2, F3, and F4 are represented by circles, squares, triangles, and diamonds, respectively. Error bars represent the standard deviation for *n* formulations, *n* = 3 for (A–D). Table S1 includes the *D*_h_ values for each formulation examined.

By conducting DLS measurements we found that the [Cho][DHP] and [Cho][OAc]-ionogel formulations exhibited relatively lower hydrodynamic diameter (*D*_h_) values compared to the [Cho][Cl]-ionogel formulations (Figure 2A–D and Table S1). Moreover, the inclusion of glycerol served to lower *D*_h_ values, in agreement with previous work.^13^ In the absence of IL, the *D*_h_ and polydispersity index (PDI) values were relatively higher for each formulation indicating enhanced aggregation propensity, which can lead to reduced structural stability.^46,47^

**Figure 3:**
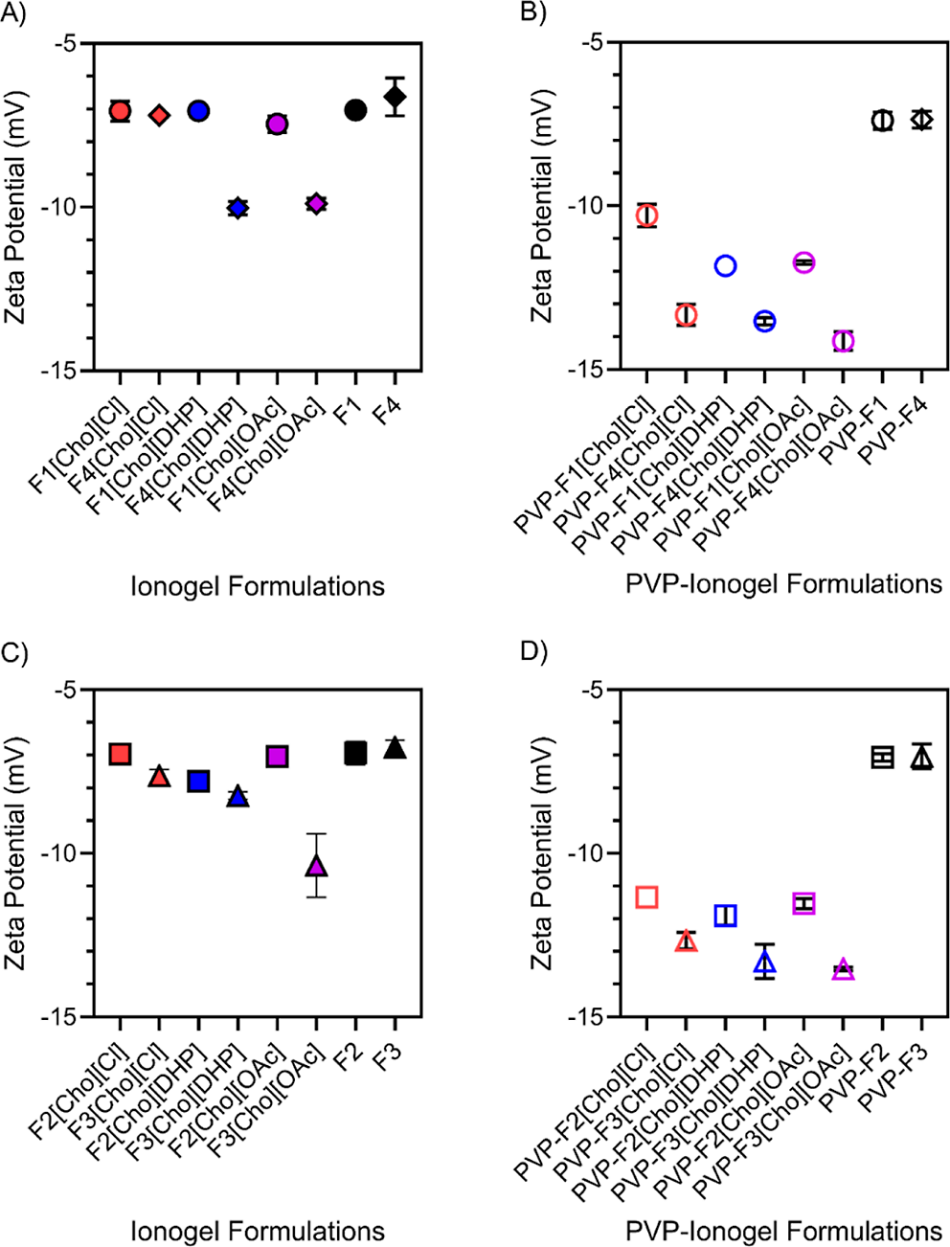
Zeta potential values for [Cho][Cl], [Cho][DHP], and [Cho][OAc]-ionogel formulations represented in red, blue, and purple, respectively. Formulations lacking IL are shown in black. F1, F2, F3, and F4 are represented by circles, squares, triangles, and diamonds, respectively. Error bars represent the standard deviation for *n* formulations, *n* = 3 for (AD). Table S1 includes the zeta potential values for each formulation examined.

We found that [Cho][DHP] and [Cho][OAc]-ionogel formulations showed relatively more negative zeta potential values compared to the [Cho][Cl]-ionogel formulations (Figure 3A–D and Table S1). PVP inclusion also resulted in more negative zeta potential values. Overall, formulations lacking IL showed less negative zeta potential values compared to the ionogel formulations. This indicated that the inclusion of IL served to reduce the aggregation propensity and induce a more negative surface charge in each system compared to formulations lacking IL, in line with previous work.^13,46,48^

### Rheological Properties of Ionogel Formulations

Rheology experiments were conducted to confirm ionogel formation under 25 °C storage and determine ionogel strength. Based on the DLS data and visual tests, ionogel formulations presenting lower *D*_h_ values and higher phenobarbital solubility were selected for examination. These included F3[Cho][DHP], F3[Cho][OAc], F4[Cho][DHP], PVP-F1[Cho][OAc], PVP-F3[Cho][OAc], and PVP-F4[Cho][DHP]. To contrast, we also examined F1[Cho][Cl], PVP-F1[Cho][Cl], and PVP-F2[Cho][Cl] showing relatively low *D*_h_ values in the absence of glycerol, yet extended periods required for ionogel formation (Tables 1 and S1).

For each system examined, we found that the storage modulus (*G*′) was greater than the loss modulus (*G*″) confirming the formation of ionogels (Figure S2).^49,50^ Upon probing the rheological properties of F1[Cho][Cl]-ionogel, requiring the longest period for ionogel formation, we found that G′ was 18 Pa. This indicated the formation of a soft ionogel. In contrast, F3[Cho][DHP] and F3[Cho][OAc], containing trehalose and glycerol, and F4[Cho][DHP], containing sucrose and glycerol, showed higher *G*′ values (52, 128, and 45 Pa, respectively) compared to the [Cho][Cl] containing formulation (Table 2). This demonstrated that in the absence of PVP, including [Cho][OAc] in the formulation resulted in a higher strength ionogel, formed over a shorter period, compared to the [Cho][Cl] and [Cho][DHP]-ionogel formulations. For the PVP containing formulations, we found that PVP-F1[Cho][Cl] and PVP-F1[Cho][OAc], containing sucrose, and PVP-F2[Cho][Cl], containing trehalose, exhibited comparable *G*′ values (25, 26 and 29 Pa, respectively). Furthermore, PVP-F3[Cho][OAc] and PVP-F4[Cho][DHP] containing glycerol exhibited the highest *G*′ values (167 and 642 Pa, respectively) of the systems examined. Overall, we found that ionogel formulations exhibiting lower *G*′ values also required an extended period for formation, and disaccharide identity was interchangeable.

**Table 2:** Maximum Storage Modulus (*G*′, Pa) for Select Phenobarbital Ionogel Formulations

ionogel formulation	maximum storage modulus (*G*′, Pa)
F1[Cho][Cl]	18
F3[Cho][OAc]	128
F3[Cho][DHP]	52
F4[Cho][DHP]	45
PVP-F1[Cho][Cl]	25
PVP-F1[Cho][OAc]	26
PVP-F2[Cho][Cl]	29
PVP-F3[Cho][OAc]	167
PVP-F4[Cho][DHP]	642

### Thermoresponsive Properties of Developed Formulations

To induce gel formation, phenobarbital formulations lacking IL were heated from 25 to 60 °C immediately following preparation. However, we failed to observe the formation of thermoresponsive gels within the examined temperature range. We observed cloudy formulations upon heating (Figure S3A) and found that the reflective cloud point (CP) values were higher for formulations containing PVP compared to formulations lacking PVP (Table 3). Notably, the silk fibroin solution, lacking excipients, presented a CP value of 49 °C, indicating that the presence of trehalose, sucrose, tween 20, histidine, and glycerol served to reduce CP values and inclusion of PVP served to raise CP values.

**Table 3:** Cloud Point Values of Phenobarbital Formulations Lacking IL

formulation	cloud point ± 2 °C
silk fibroin solution	49
F1	44
F2	45
F3	47
F4	48
PVP-F1	51
PVP-F2	52
PVP-F3	54
PVP-F4	59

**Figure 4:**
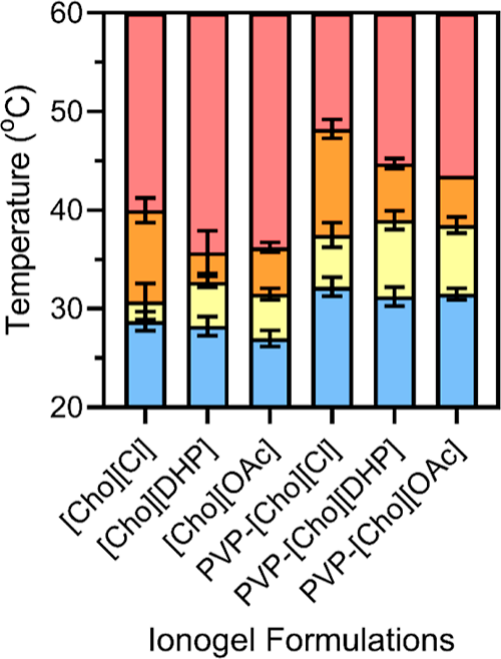
Visual transition states of [Cho][OAc], [Cho][DHP], and [Cho][Cl]-ionogel formulations in the absence and presence of PVP, heated from 25 to 60 °C at 1 °C intervals. As temperature was increased, the IL containing formulations transitioned from transparent formulations (blue) to transparent viscous ionogel-like formulations (yellow), to cloudy viscous ionogel-like formulations (orange) and finally to cloudy ionogels (red). The mean average values are shown for each transition and error bars represent the standard deviation for *n* formulations (*n* = 4).

**Figure 5:**
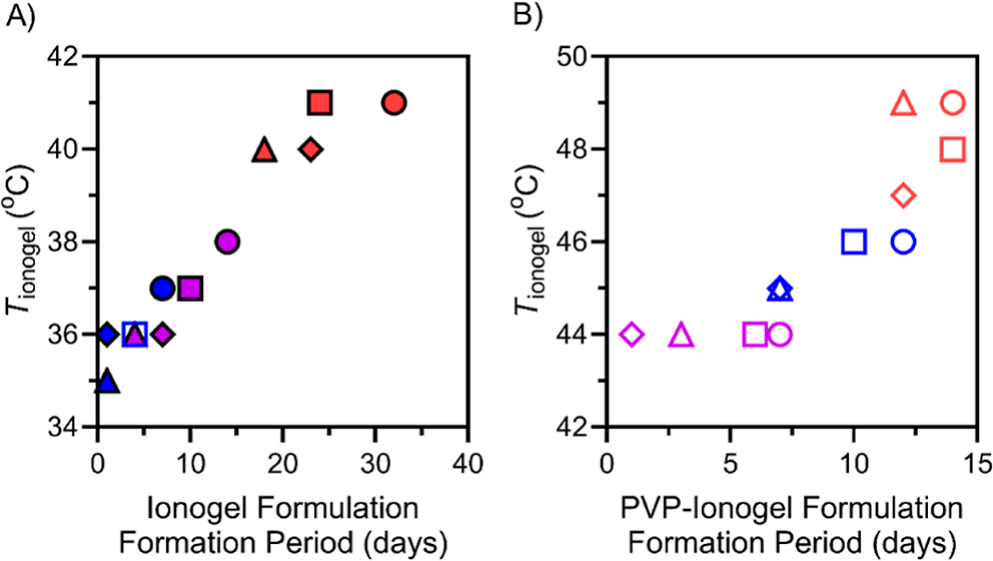
Correlation of the onset temperature for ionogel formation (*T*_ionogel_) and ionogel formation period under 25 °C storage. [Cho][Cl], [Cho][DHP], and [Cho][OAc]-ionogel formulations are shown in red, blue, and purple, respectively. F1, F2, F3, and F4-ionogel formulations are represented by circles, squares, triangles, and diamonds, respectively.

In contrast, upon heating the IL containing formulations we observed multiple phase transitions for each system, from transparent formulations to transparent viscous ionogel-like formulations, followed by cloudy viscous ionogel-like formulations and finally the formation of cloudy ionogels (Figures 4 and S3B). In the absence of PVP, the onset temperature for ionogel formation (*T*_ionogel_) ranged between 35 and 41 °C, decreasing in the order of [Cho][Cl], [Cho][DHP], and [Cho][OAc] (Table S2). In comparison, PVP-ionogel formulations showed higher *T*_ionogel_ values ranging between 44 and 49 °C. Notably, the PVP-[Cho][DHP] and PVP-[Cho][OAc]-ionogels presented similar and relatively lower *T*_ionogel_ values compared to the PVP-[Cho][Cl]-ionogels. We observed that the thermoresponsive ionogels, formed via heating, maintained structural integrity and lacked phase separation following cooling to 25 °C and under 25 °C storage over two months. Notably, we found that ionogels exhibiting lower *T*_ionogel_ values also required reduced periods for formation under 25 °C storage (Figure 5).

### Morphology of Developed Formulations

**Figure 6:**
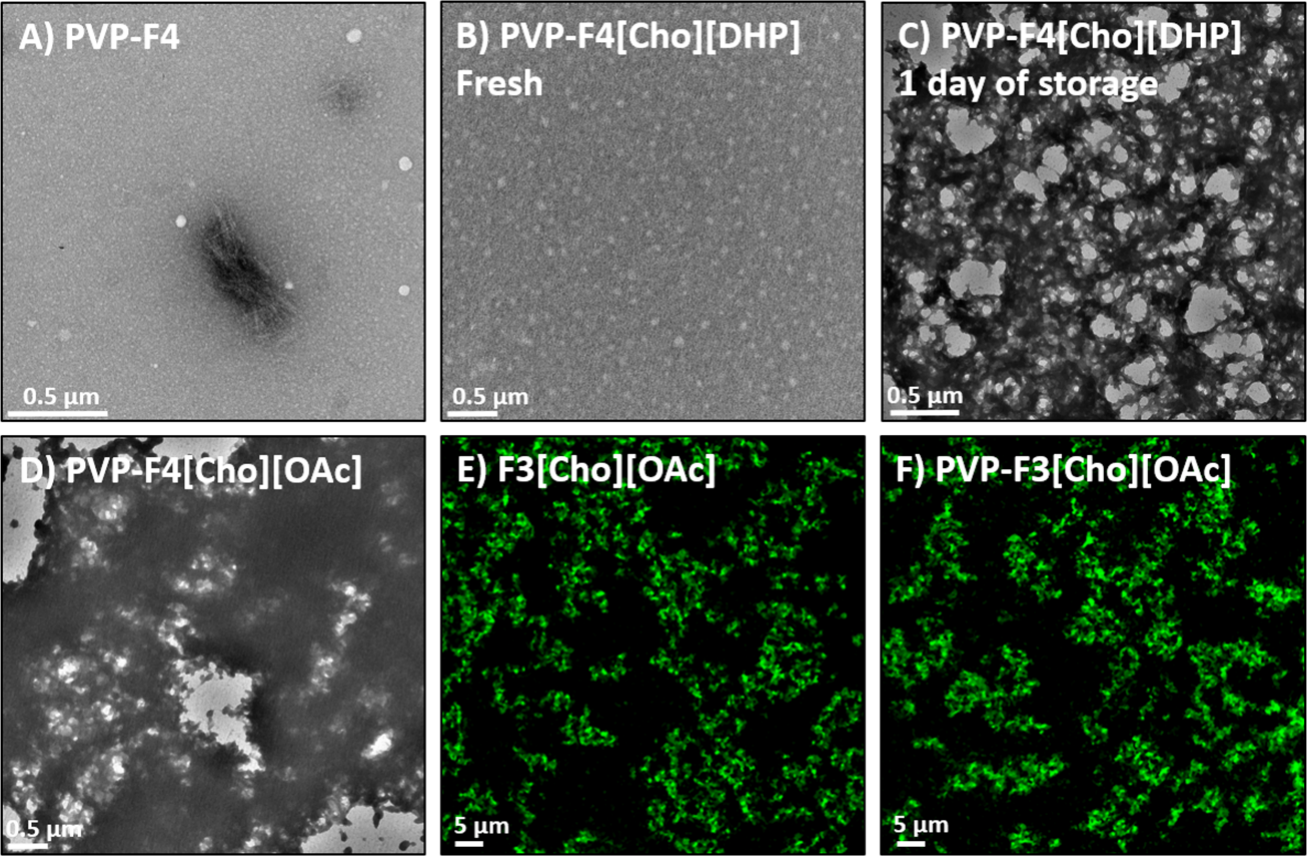
Negative staining TEM images of systems stored under 25 °C; (A) PVP-F4, (B) PVP-F4[Cho][DHP] immediately following preparation and (C) following 1 day under 25 °C storage, and (D) PVP-F4[Cho][OAc]-ionogel following 7 days under 25 °C storage. CRM images of systems immediately following heating from 25 to 60 °C; (E) F3[Cho][OAc] and (F) PVP-F3[Cho][OAc]-ionogels. For additional formulations see Figures S4 and S5.

We performed negative staining TEM experiments to visualize the morphologies of ionogels formed under 25 °C storage. Systems of enhanced phenobarbital solubility, lower *D*_h_ values, and higher strengths were selected for examination. We demonstrate that under 25 °C storage, PVP-F4, lacking IL, exhibited fibrillar aggregates as opposed to a gel network (Figure 6A). This is in agreement with our visual observations of formulations lacking IL failing to show gel formation (Figure S4A). We also demonstrate that PVP-F4[Cho][DHP] examined immediately following preparation failed to exhibit an ionogel network (Figure 6B). However, following 1 day of storage under 25 °C, we observed a densely porous ionogel network (Figure 6C). The PVP-F4[Cho][OAc]-ionogel network appeared to be of a lower degree of porosity (Figure 6D). Furthermore, in the absence of PVP we find that the inclusion of [Cho][DHP] and [Cho][OAc] resulted in the formation of dense networks (Figure S4B–D). Additionally, we performed CRM to immediately examine the morphologies of ionogels formed via heating and also observed densely porous ionogel networks (Figures 6E,F and S5A–C).

Overall, the TEM and CRM experiments demonstrate that interchanging the disaccharide component in the formulation results in negligible effects on ionogel morphology; however, the inclusion of diverse ILs results in greater distinctions between ionogel morphologies. Notably, these trends are also consistent with our findings from the rheology experiments.

### Energetic Properties and Hydrogen Bond Networks of Developed Systems

We performed DFT calculations to gain insight into the energetic properties and nature of hydrogen bonding of the diverse ionogel formulations. The examined systems consisted of a given IL ion pair, cation and anion, each integrated with a silk fibroin AGAGA polypeptide, trehalose, sucrose and combined trehalose and glycerol molecules, and a dimeric PVP unit (Table 4).

**Table 4:** Change in Gibbs Free Energy (ΔG), Enthalpy (Δ*H*), and Total Interaction Energy (Δ*E*) of Developed Systems

formulation ingredients	ionic liquids	Δ*G* (kcal/mol)	Δ*H* (kcal/mol)	Δ*E* (kcal/mol)
	[Cho][Cl]	–13.5	–28.2	–24.7
trehalose	[Cho][DHP]	–14.7	–33.0	–27.2
	[Cho][OAc]	–10.8	–28.8	–25.1
	[Cho][Cl]	–12.1	–24.4	–22.6
sucrose	[Cho][DHP]	–17.8	–34.7	–29.6
	[Cho][OAc]	–11.6	–29.1	–24.8
	[Cho][Cl]	–10.8	–23.2	–21.4
trehalose and glycerol	[Cho][DHP]	–16.1	–30.8	–26.9
	[Cho][OAc]	–12.5	–25.1	–22.9
	[Cho][Cl]	–7.5	–19.5	–17.4
silk fibroin	[Cho][DHP]	–11.3	–26.0	–22.7
	[Cho][OAc]	–6.3	–20.3	–19.0
	[Cho][Cl]	–12.5	–25.0	–22.9
PVP	[Cho][DHP]	–0.8	–15.1	–11.8
	[Cho][OAc]	–8.5	–23.3	–21.4

For each examined system we found that the values obtained for the change in Gibbs free energy (Δ*G*), enthalpy (Δ*H*), and total interaction energy (Δ*E*) were negative. This indicated the formation of energetically stable systems. We found that for the silk fibroin chains, trehalose, sucrose, and combined trehalose and glycerol molecules, the lowest Δ*G*, Δ*H,* and Δ*E* values were presented upon inclusion of [Cho][DHP] followed by [Cho][Cl] and [Cho][OAc]. In contrast, the dimeric PVP unit combined with [Cho][Cl] showed the lowest Δ*G*, Δ*H,* and Δ*E* values compared to the [Cho][OAc] and [Cho][DHP] containing systems.

To further rationalize these trends, we calculated the Kamlet–Taft β parameters and applied the quantum theory of atoms in molecules (QTAIM) framework (Table S3) to probe the intermolecular hydrogen bonding for each system. We found that the [Cho][OAc] inclusive systems exhibited the strongest intermolecular hydrogen bonding interactions of the systems examined. This is consistent with our calculated Kamlet–Taft β parameters for acetate, dihydrogen phosphate, and chloride anions found to be 1.24, 0.89, and 0.86, respectively. We observed that the dihydrogen phosphate anion acts as a hydrogen bond donor and acceptor, and accordingly facilitates multiple strong intermolecular hydrogen bonding interactions. The acetate and chloride anions act solely as hydrogen bond acceptors, thus relatively limiting the number of intermolecular hydrogen bonds formed. Specifically, the chloride anion facilitates the formation of the weakest intermolecular hydrogen bonding interactions, explaining the least negative zeta potential values found for the [Cho][Cl]-ionogel formulations.

## Discussion

We rationalize that we failed to observe gel formation in the absence of IL since the excipient components imparted limited structural stabilization and macromolecular crowding of the silk fibroin chains. Notably, we observed the formation of aggregates, likely involving intermolecular hydrogen bonding interactions between the silk fibroin chains and excipient molecules and intramolecular hydrogen bonding interactions between the silk fibroin chains.^27,30,51,52^ In particular, intermolecular hydrogen bonding interactions between the pendant pyridine groups of the PVP molecules and the amino acid residues of the silk fibroin chains could promote aggregate formation,^52,53^ in agreement with the DLS data and microscopy observations. These aggregates could be of higher thermal stability compared to the aggregates formed in the absence of PVP, consistent with our observed CP values.

We suggest that IL inclusion promotes the formation of diverse ionogels via electrostatic, inter- and intramolecular hydrogen bonding interactions.^8,54,55^ We consider that for the [Cho][Cl]-ionogel formulations, of highest ionic strength, [Cho][Cl] incorporation could result in surface charge accumulation on the silk fibroin chains and weak intermolecular hydrogen bonding interactions.^13,56−61^ This would explain our findings of higher *T*_ionogel_ values, lower phenobarbital solubility, and impeded [Cho][Cl]-ionogel formation. In contrast, the extended structure of the dihydrogen phosphate anions could contribute to the formation of strong intermolecular hydrogen bonding networks and steric effects.^62−65^ This aligns with our observations of a shorter formation period, greater strength, structural and energetic stability for the [Cho][DHP]-ionogels compared to the [Cho][Cl]-ionogels. The acetate anions possess more compact structures relative to the dihydrogen phosphate anions, and the ionic strength of the [Cho][OAc]-ionogel formulations is lower compared to the [Cho][Cl]-ionogel formulations. Accordingly, we propose that the [Cho][OAc]-ionogels exhibit strong intermolecular hydrogen bonding interactions, limited steric hindrance, electrostatic stabilization, and the greatest macromolecular confinement effects. This is consistent with the shortest formation period, highest strength, and greatest structural and energetic stability found for the [Cho][OAc]-ionogels lacking PVP.

Logically, the degree of intermolecular hydrogen bonding interactions, macromolecular crowding, and confinement effects would be greater for formulations containing PVP molecules. Overall, this explains the higher *T*_ionogel_ values and reduced formation period for the PVP-ionogel formulations. We suggest that the IL ions penetrate the silk fibroin chains, serving as anchoring units for the hydrophilic PVP molecules to diffuse and form strong intermolecular hydrogen bonding interactions between the silk fibroin chains.^66,67^ Synergistically, greater macromolecular confinement and electrostatic interactions would also contribute to the higher structural and energetic stabilization of the PVP-ionogel formulations. We propose that for the PVP-[Cho][Cl]-ionogels, the compact structure of [Cho][Cl] could permit limited anchoring and delayed diffusion of the PVP molecules in the silk fibroin chains. As a result, ionogel formation would be delayed and *T*_ionogel_ values would be raised, as we observed. In contrast, the relatively extended structure of [Cho][OAc] could provide more effective anchoring and diffusion of the PVP molecules, thus reducing the required formation period for PVP-[Cho][OAc]-ionogels. Following this logic, [Cho][DHP] could most effectively anchor to the silk fibroin chains and facilitate the diffusion of the PVP molecules, resulting in enhanced macromolecular confinement effects. Furthermore, potential steric effects of the extended structure of the dihydrogen phosphate anions would be compensated for, and the structural and energetic stabilization of the PVP-[Cho][DHP]-ionogels would be increased.^64−67^ This aligns with our finding of strong intermolecular hydrogen bonding interactions, greatest strength, and least delayed period for the formation of the PVP-[Cho][DHP]-ionogel formulations containing glycerol. The inclusion of glycerol molecules, containing multiple hydroxyl groups, likely enhanced the intermolecular hydrogen bonding interactions between the silk fibroin chains, disaccharides, surfactants, and amino acid molecules, in the absence and presence of PVP.^2,64−69^ Thus, glycerol containing formulations could exhibit strong intra- and intermolecular hydrogen bonds, and enhanced macromolecular crowding and confinement effects, yielding the formation of ionogels of greater strength, and higher structural and energetic stability.

## Conclusions

We demonstrate a rational systematic approach for the development of thermoresponsive silk fibroin-based ionogels, offering multiple advantages including formulation simplicity, enhanced drug solubility, and a range of physicochemical, rheological and energetic properties that can be highly controlled. Under 25 °C storage, overall, we observed that [Cho][DHP] and [Cho][OAc]-ionogel formulations formed relatively rapidly, shortest following 1 day, compared to [Cho][Cl]-ionogel formulations requiring extended formation periods, as long as 32 days. Additionally, upon heating the fresh aqueous IL inclusive formulations from 25 to 60 °C, each system displayed thermoresponsive properties. In particular, [Cho][DHP] and [Cho][OAc]-ionogels lacking PVP presented *T*_ionogel,_ values below 37 °C, and hence could be beneficial for local and controlled drug delivery applications. Furthermore, PVP-[Cho][DHP]-ionogel formulations containing glycerol exhibited the highest strength, 642 Pa. In contrast, formulations lacking IL showed reduced drug solubility, and we failed to observe gel formation over two months under 25 °C storage nor upon heating to 60 °C. On the basis of our experimental and computational results, we suggest that the dihydrogen phosphate anions act as hydrogen bond donors and acceptors, facilitating a network of multiple strong intermolecular hydrogen bonding interactions, linked to higher ionogel strength and rapid formation. Given that the acetate anions act solely as hydrogen bond acceptors, the number of intermolecular hydrogen bonds is relatively reduced, resulting in lower ionogel strength. The chloride anions facilitate the formation of the weakest intermolecular hydrogen bonding interactions, in line with the delayed formation of [Cho][Cl]-ionogels. Moreover, glycerol and PVP inclusion could contribute to strong intermolecular hydrogen bonding, increasing macromolecular crowding and confinement effects, overall yielding ionogels of greater strength, structural and energetic stability. Future work should continue to explore the balance of energetic forces driving the formation of hydrogen bonds in ionogel systems and the relationship between the intermolecular interactions, stability, and drug release behavior of diverse ionogels. As increasingly advanced ionogel designs are explored, compiling this information will be critical to the realization and clinical translation of ionogel platforms for drug delivery applications.

## Materials and Methods

### Materials

[Cho][Cl], [Cho][OAc], sucrose, trehalose, l-histidine, tween 20, glycerol, PVP, phenobarbital, and solution of silk fibroin derived from the domesticated *Bombyx mori* silkworm were purchased from Sigma-Aldrich Co. Ltd. (Gillingham, Dorset, UK). [Cho][DHP] was purchased from IoLiTec-Ionic Liquids Technologies GmbH (Heilbronn, Germany). All ingredients were stored as recommended and used without further purification.

### Preparation of Ionogel Formulations and Formulations Lacking IL

Stock solutions of [Cho][DHP], [Cho][OAc], and [Cho][Cl] were prepared as previously described.^63^Table 5 shows the composition of the formulation buffers developed herein. For each, stock solutions were prepared. The specified components were dissolved in ultrapure water (ELGA LabWater, High Wycombe, UK) in a glass vial (Thermo Fisher Scientific Inc., Waltham, MA, USA) and mixed by means of vortex and sonication for 30 min at 25 °C.

**Table 5:** Designed Formulation Buffers with Each Component Expressed as % w/w

formulation	trehalose	Tween 20	sucrose	glycerol	histidine
**F1**		0.03	65		4
**F2**	65	0.03			4
**F3**	65	0.03		3	4
**F4**		0.03	65	3	4

Ionogel formulations were prepared by stirring each IL with silk fibroin solution at 25 °C, and the desired formulation buffer (Table 5) was added dropwise to achieve a silk fibroin/formulation buffer/IL ratio of 1:1.6:12.8, by weight. In the absence of IL, systems contained a silk fibroin/formulation buffer ratio of 1:1.6 by weight and prepared as before. To produce the PVP inclusive formulations, silk fibroin and PVP were mixed at a ratio of 1:1 by weight, and the desired formulation buffer in the presence and absence of IL was added as before. Each system developed herein consisted of 4 mg/mL of phenobarbital. Sodium hydroxide and hydrochloric acid were added dropwise to obtain a formulation pH of 6.5; ensured by pH measurement with the pH electrode Mettler Toledo InLab Micro (Wolflabs, Pocklington, York, UK).

### Visual Tests

Visual tests were conducted as previously described^13^ to determine phenobarbital solubility, the CP and *T*_ionogel_ values for the formulation. The CP value was indicative of the onset temperature at which the transparent formulation first became cloudy upon heating from 25 to 60 °C. The *T*_ionogel_ value was indicative of the onset temperature at which a cloudy ionogel formed, defined as lack of flow upon tube inversion.

### DLS and Zeta Potential Measurements

We performed DLS measurements employing a Zetasizer μV instrument (Malvern Panalytical Ltd., Malvern, UK). Each aqueous formulation, lacking and containing IL, was measured immediately following preparation, undiluted and in a disposable cuvette (Malvern Panalytical Ltd., Malvern, UK). Following 60 s of equilibration time, each formulation was measured in triplicate at 25 °C at a 90° scattering angle to obtain the mean *D*_h_ and PDI values reported.

For each fresh undiluted aqueous formulation, zeta potential measurements were performed employing a Litesizer 500 (Anton Paar GmbH, Ostfildern, Germany). Formulations were injected into Omega cuvettes (Anton Paar GmbH, Ostfildern, Germany) and inserted in the device measuring chamber. For each formulation, measurements were performed in triplicate using automatic mode, at 25 °C following 60 s of equilibration time, to obtain the mean zeta potential values reported.

### Negative Staining TEM and CRM Experiments

For TEM experiments, for each examined sample, a 4 μL droplet was spotted onto a Formavar/carbon coated copper grid (300 mesh) (Agar Scientific Ltd., Stansted, Essex, UK) for 1 min. Excess sample was removed by blotting dry with Whatman filter paper (Sigma-Aldrich Co. Ltd., Gillingham, Dorset, UK) after which the grid was washed with water. Finally, the grid was stained with 2% w/v uranyl acetate (Agar Scientific Ltd., Stansted, Essex, UK). Samples were imaged on a T12 Spirit electron microscope (Thermo Fisher Scientific Inc., Hillsboro, OR, USA).

For CRM experiments, unlabeled samples were mounted on MatTek glass bottom dishs (MatTek Life Sciences, Ashland, MA, USA) and imaged employing a Leica Stellaris 8 TCS-SP2 (Leica Microsystems GmbH, Wetzlar, Germany) equipped with an HC PL APO 63x/1.40 NA OIL CS2 objective. A laser at wavelength 491 nm was used to illuminate each sample and the reflected light was detected in the range of 481–498 nm. Images were processed and analysed using Fiji (NIH, Bethesda, MD, USA).

### Rheology Experiments

The rheological properties of the ionogels formed under 25 °C storage were examined, as previously reported.^13^ Briefly, rheology experiments were performed using a TA Discovery HR-1 hybrid rheometer (TA Instruments, Elstree, Hertfordshire, UK) equipped with a 40 mm parallel steel plate (996921) and a solvent trap. Temperature ramp measurements were conducted from 25 to 50 °C at 1 °C/min with the angular frequency (ω) fixed at 1 rad/s and strain (γ) fixed at 1%. The storage modulus and loss modulus (*G*′ and *G*″, respectively) were measured, and in the case of ionogel formation G′ exceeds G″.

### DFT Calculations and Analysis

DFT calculations were carried out with the Gaussian 16 (revision C.01) suite of programs.^70^ All structures were fully optimized under no symmetry constraints and confirmed as minima at the ωB97XD/6-311++g(d,p) level of theory. Optimization convergence criteria were set to 10^–9^ on the density matrix and 10^–7^ on the energy matrix. The numerical integration grid was set to a pruned (optimized) grid of 99 radial shells and 590 angular points per shell. These criteria were maintained for the vibrational and counterpoise calculations. Vibrational frequencies and zero-point vibrational energy corrections (ZPE) were obtained within the harmonic approximation for each structure, and basis set superposition error (BSSE) was computed using the counterpoise method.^71^ Δ*G*, Δ*H,* and Δ*E* values reported for the IL inclusive formulations were obtained using the following equation
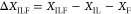
where *X* represents the BSSE corrected enthalpy (*H*), Gibbs free energy (*G*), or total interaction energy (*E*). Analysis of the electron density was performed within the QTAIM framework employing the AIMALL package,^72^ as per previous investigations into noncovalent interactions in ILs.^73,74^ Kamlet–Taft β parameters were calculated for the acetate, dihydrogen phosphate, and chloride anions, as previously described.^75^

## Data Availability

All data required to evaluate the conclusions in the manuscript are present in the main manuscript and/or the Supporting Information. Additional data related to the manuscript may be requested from the authors.

## Supporting Information Available

Photographs of phenobarbital in silk fibroin solution, polyvinylpyrrolidone (PVP)-F3[Cho][OAc] fresh and following 7 days under 25 °C storage, *D*_h_, PDI, and zeta potential values for each ionogel formulation and formulations lacking IL, storage and loss moduli as a function of temperature (°C) for PVP-F1[Cho][Cl], F1-[Cho][Cl], F4[Cho][DHP], PVP-F2[Cho][Cl], PVP-F1[Cho][OAc], F3[Cho][DHP], PVP-F4[Cho][DHP], PVP-F3[Cho][OAc], and F3[Cho][OAc]-ionogels, *T*_ionogel_ values obtained upon heating each ionogel formulation from 25 to 60 °C, photographs of PVP-F3 and PVP-F3[Cho][OAc]-ionogel following heating from 25 to 60 °C, negative staining TEM images of silk fibroin solution, F3[Cho][OAc], F4[Cho][OAc], and F3[Cho][DHP]-ionogels formed under 25 °C storage, CRM images of F3[Cho][DHP], F4[Cho][DHP], and PVP-F4[Cho][DHP]-ionogels formed via heating from 25 to 60 °C, and QTAIM hydrogen bonding parameters, electron density, Laplacian, kinetic electron energy density, potential electron energy density, and total electron energy density calculated for the intermolecular hydrogen bonding interactions of ([Cho][DHP]), ([Cho][OAc]), and ([Cho][Cl]) combined with glycerol, trehalose, sucrose, trehalose and glycerol, and the dimeric PVP unit (PDF)
