# Supplementary material for: Unveiling the Rational Development of Stimuli-Responsive Silk Fibroin-Based Ionogel Formulations
Source: Chem Mater. 2023 Jul 20;35(15):5798–808. doi: 10.1021/acs.chemmater.3c00303 (PMC10413859; doi:10.1021/acs.chemmater.3c00303)
Supplement: Supplementary file 1 — cm3c00303_si_001.pdf [file cm3c00303_si_001.pdf]

Unveiling the Rational Development of Stimuli-Responsive Silk Fibroin-Based Ionogel Formulations

Talia A. Shmool,[a,*] Laura K. Martin,[b] Andreas Jirkas,[a] Richard P. Matthews,[ac] Anna P. Constantinou,[d] Devkee M. Vadukul,[e] Theoni K. Georgiou,[d] Francesco A. Aprile,[ef] Jason P. Hallett [a,*]

[a] Department of Chemical Engineering, Imperial College London, South Kensington Campus, London SW7 2AZ, UK

[b] Department of Engineering Science, University of Oxford, Parks Road, Oxford OX1 3PJ, UK

[c] Department of Bioscience, School of Health, Sports and Bioscience, University of East London, Stratford, London E15 4LZ, UK

[d] Department of Materials, Imperial College London, South Kensington Campus, London SW7 2AZ, UK

[e] Department of Chemistry, Molecular Sciences Research Hub, Imperial College London, London W12 0BZ, UK

[f] Institute of Chemical Biology, Molecular Sciences Research Hub, Imperial College London, London W12 0BZ, UK

***E-mail:** t.shmool20@imperial.ac.uk, j.hallett@imperial.ac.uk **Tel:** +44 (0)20 7594 5388

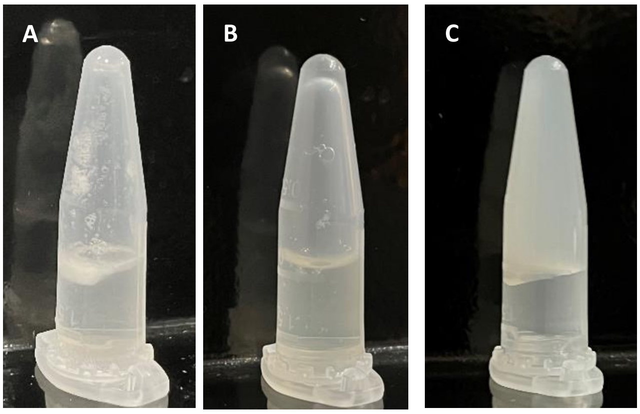

Figure S1. Photographs of phenobarbital in A) silk fibroin solution, B) polyvinylpyrrolidone (PVP)-F3[Cho][OAc] fresh and C) on seventh day under 25 °C storage. The yellow tinge of B and C is due to the nature of the PVP ingredient.

Table S1. Hydrodynamic diameter (D_h), polydispersity index (PDI) and zeta potential values for each Ionogel formulation and formulations lacking ionic liquid (IL).

System	D_h	PDI	Zeta potential
Silk fibroin solution	477 ± 10	0.91 ± 0.08	-7.0 ± 0.4
F1	567 ± 4	0.83 ± 0.3	-7.0 ± 0.1
F2	536 ± 2	0.9 ± 0.2	-7.6 ± 0.07
F3	362 ± 3	0.64 ± 0.04	-6.7 ± 0.3
F4	369 ± 10	0.95 ± 0.07	-6.8 ± 0.7
PVP-F1	556 ± 21	0.4 ± 0.1	-7.0 ± 0.5
PVP-F2	495 ± 5	0.7 ± 0.2	-7.0 ± 0.4
PVP-F3	456 ± 6	0.71 ± 0.2	-7.0 ± 0.4
PVP-F4	378 ± 4	0.97 ± 0.04	-7.4 ± 0.3
F1[Cho][Cl]	350 ± 3	0.30 ± 0.01	-7.1 ± 0.3

F1[Cho][DHP]	333 ± 4	0.28 ± 0.007	-7.1 ± 0.3
F1[Cho][OAC]	317 ± 3	0.27 ± 0.008	-7.5± 0.2
PVP-F1[Cho][Cl]	339 ± 1	0.28 ± 0.02	-10 ± 0.3
PVP-F1[Cho][DHP]	309 ± 4	0.29 ± 0.05	-12 ± 0.2
PVP-F1[Cho][OAC]	324 ± 4	0.26 ± 0.02	-12 ± 0.06
F2[Cho][Cl]	305 ± 2	0.27 ± 0.01	-7.0 ± 0.06
F2[Cho][DHP]	313 ± 3	0.27 ± 0.004	-7.8 ± 0.2
F2[Cho][OAC]	284 ± 1	0.25 ± 0.03	-7.0 ± 0.06
PVP-F2[Cho][Cl]	320 ± 3	0.26 ± 0.01	-11 ± 0.3
PVP-F2[Cho][DHP]	310 ± 3	0.28 ± 0.06	-12 ± 0.3
PVP-F2[Cho][OAC]	337 ± 3	0.27 ± 0.01	-12 ± 0.2
F3[Cho][Cl]	293 ± 3	0.27 ± 0.02	-7.6 ± 0.2
F3[Cho][DHP]	280 ± 2	0.26 ± 0.05	-8.2 ± 0.1
F3[Cho][OAC]	267 ± 6	0.29 ± 0.06	-10 ± 1
PVP-F3[Cho][Cl]	318 ± 3	0.27 ± 0.02	-13 ± 0.3
PVP-F3[Cho][DHP]	235 ± 2	0.23 ± 0.1	-13 ± 0.5
PVP-F3[Cho][OAC]	296 ± 3	0.25 ± 0.03	-14 ± 0.06
F4[Cho][Cl]	302 ± 2	0.28 ± 0.04	-7.3 ± 0.1
F4[Cho][DHP]	273 ± 4	0.28 ± 0.01	-10 ± 0.3
F4[Cho][OAC]	271 ± 2	0.27 ± 0.03	-10 ± 0.2
PVP-F4[Cho][Cl]	305 ± 3	0.30 ± 0.07	-13 ± 0.3
PVP-F4[Cho][DHP]	276 ± 2	0.31 ± 0.04	-14 ± 0.1
PVP-F4[Cho][OAC]	282 ± 5	0.27 ± 0.06	-14 ± 0.3

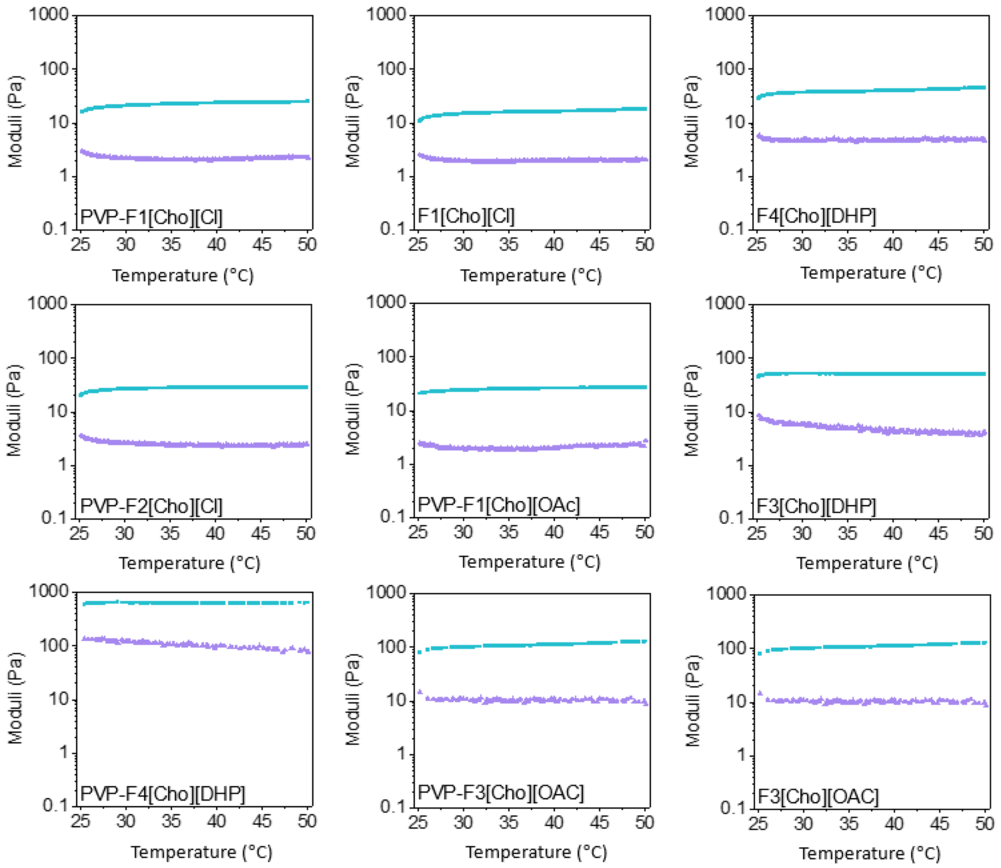

Figure S2. Storage (G′, cyan squares) and loss (G″, purple triangles) moduli as a function of temperature (°C) for select Ionogel formulations.

Table S2. Onset temperature for Ionogel formation (T_{ionogel}) upon heating from 25 to 60 °C.

**Ionogel formulation**	$\boldsymbol{T_{\text{ionogel}}}$ **(°C)**
F1[Cho][OAc]	37
F1[Cho][DHP]	38
F1[Cho][Cl]	41
F2[Cho][OAc]	36
F2[Cho][DHP]	37
F2[Cho][Cl]	41
F3[Cho][OAc]	35
F3[Cho][DHP]	36
F3[Cho][Cl]	40
F4[Cho][OAc]	36
F4[Cho][DHP]	36
F4[Cho][Cl]	40
PVP-F1[Cho][OAc]	46
PVP-F1[Cho][DHP]	44
PVP-F1[Cho][Cl]	49
PVP-F2[Cho][OAc]	46
PVP-F2[Cho][DHP]	44
PVP-F2[Cho][Cl]	48
PVP-F3[Cho][OAc]	45
PVP-F3[Cho][DHP]	44

PVP-F3[Cho][Cl]	49
PVP-F4[Cho][OAc]	45
PVP-F4[Cho][DHP]	44
PVP-F4[Cho][Cl]	47

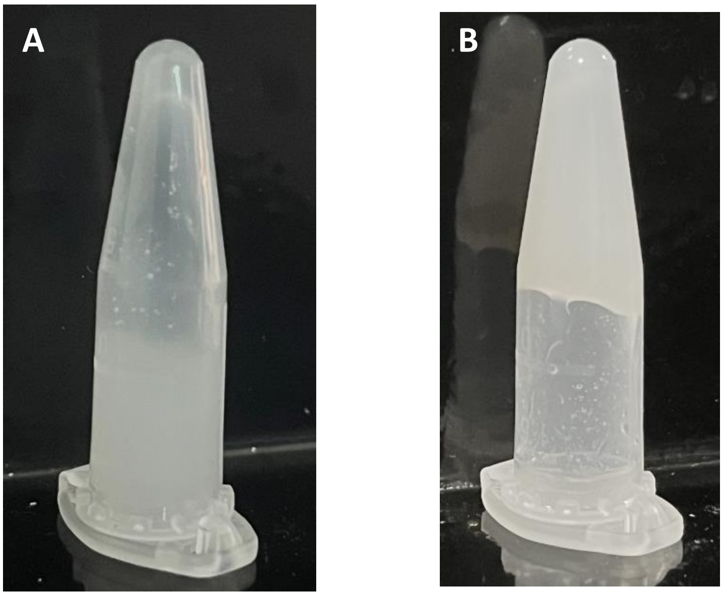

Figure S3. Photographs of A) PVP-F3 and B) PVP-F3[Cho][OAc]-Ionogel following heating from 25 to 60 °C.

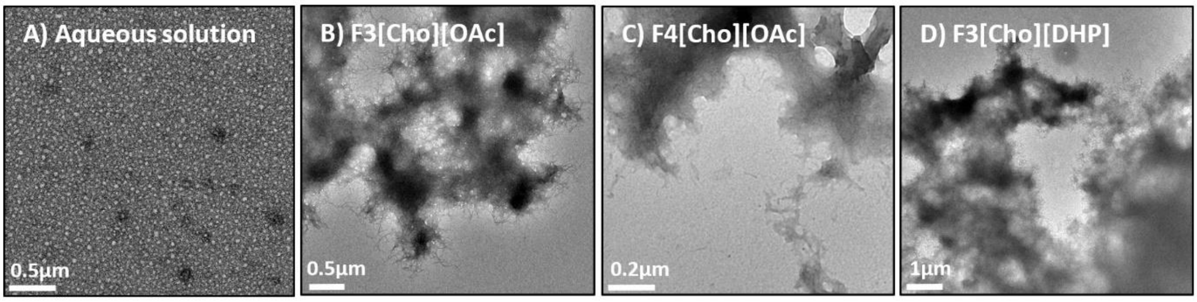

Figure S4. Negative staining transmission electron microscopy images of A) silk fibroin aqueous solution, B) F3[Cho][OAc], C) F4[Cho][OAc] and D) F3[Cho][DHP]-Ionogels formed under 25 °C storage.

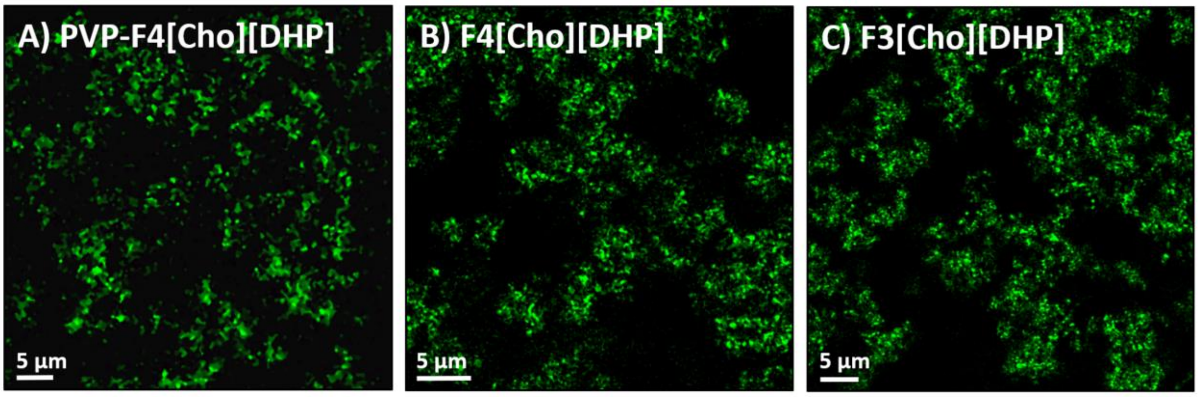

Figure S5. Confocal reflection microscopy images of A) PVP-F4[Cho][DHP], B) F4[Cho][DHP], C) F3[Cho][DHP]-Ionogels formed via heating from 25 to 60 °C.

Table S3. QTAIM hydrogen bonding parameters electron density, ρ(r) , Laplacian, $\nabla^2 r$(r), kinetic electron energy density, *V*(r), potential electron energy density, *G*(r) and total electron energy density, *H*(r), calculated for the intermolecular hydrogen bonding interactions of a given ionic liquid. These include choline dihydrogen phosphate ([Cho][DHP]), choline acetate ([Cho][OAc]), choline chloride ([Cho][Cl]) combined with glycerol (Gly), trehalose (Tre), sucrose (Suc), trehalose and glycerol and the dimeric PVP unit. The subscripts a and b are representative of the distinct hydrogen bond donor sites of the specified molecular and ionic species. The subscripts c and d are representative of the distinct hydrogen bond acceptor sites of the specified molecular and ionic species. A lack of subscript indicates a singular hydrogen bond acceptor and donor units.

Ionic liquid	Formulation ingredient	Hydrogen bond donor	Hydrogen bond acceptor	ρ (r) / au	$\nabla^2 r$ (r) / au	*V* (r) / au	*G* (r) / au	*H* (r) / au
[Cho][Cl]	Glycerol	(Cho)OH	Cl^-	0.0237	0.0654	-0.0153	0.0158	0.0005
		(Gly)OH_a	Cl^-	0.0265	0.0699	-0.0177	0.0176	-0.0001
		(Gly)OH_b	Cl^-	0.0276	0.0711	-0.0188	0.0183	-0.0005
	Trehalose	(Cho)OH	Cl^-	0.0297	0.0728	-0.021	0.0196	-0.0014
		(Tre)OH_a	Cl^-	0.0296	0.0745	-0.0211	0.0198	-0.0013
		(Tre)OH_b	(Cho)O_c	0.0352	0.1257	-0.0312	0.0313	0.0001
	Trehalose and Glycerol	(Cho)OH	Cl^-	0.0257	0.07	-0.0174	0.0175	0.0001
		(Tre)OH_a	(Gly)O_c	0.0333	0.123	-0.0294	0.0301	0.0007
		(Tre)OH_b	Cl^-	0.0256	0.0712	-0.0174	0.0176	0.0002
		(Gly)OH_a	Cl^-	0.0224	0.0644	-0.0143	0.0152	0.0009
		(Gly)OH_b	Cl^-	0.022	0.0637	-0.0137	0.0148	0.0011
	Sucrose	(Cho)OH	Cl^-	0.0220	0.0622	-0.0138	0.0147	-0.0009
		(Suc)OH_a	Cl^-	0.0370	0.0803	-0.0290	0.0254	-0.0036
		(Suc)OH_b	Cl^-	0.0189	0.0558	-0.0109	0.0124	0.0015
	PVP	(Cho)OH	(PVP)O_c	0.0349	0.1328	-0.0318	0.0325	0.0007
[Cho][DHP]	Glycerol	(Cho)OH	(DHP)O_c	0.0466	0.157	-0.0473	0.0433	-0.0040
		(Gly)OH_a	(DHP)O_d	0.0515	0.1581	-0.0528	0.0462	-0.0066
		(Gly)OH_b	(Cho)O_c	0.0296	0.1018	-0.0237	0.0246	0.0009
	Trehalose	(Cho)OH	(DHP)O_c	0.0273	0.0984	-0.0221	0.0234	0.0013
		(Tre)OH_a	(DHP)O_c	0.027	0.1023	-0.022	0.0238	0.0018
		(Tre)OH_b	(DHP)O_d	0.0371	0.1343	-0.0344	0.034	-0.0004
		(DHP)OH	(Tre)O_c	0.033	0.118	-0.0292	0.0293	0.0001

	Trehalose and Glycerol	(Cho)OH	(DHP)O$_c$	0.0256	0.0941	-0.0206	0.0221	0.0015
		(Tre)OH$_a$	(DHP)O$_c$	0.0388	0.1363	-0.0363	0.0352	-0.0011
		(DHP)OH	(Tre)O$_c$	0.0317	0.1183	-0.0275	0.0285	0.0010
		(Tre)OH$_b$	(Gly)O$_c$	0.0278	0.1046	-0.0228	0.0245	0.0017
		(DHP)OH	(Gly)O$_c$	0.0386	0.1276	-0.0351	0.0335	-0.0016
		(Gly)OH$_a$	(DHP)O$_d$	0.0396	0.1287	-0.0358	0.034	-0.0018
		(Gly)OH$_b$	(DHP)O$_d$	0.0332	0.12	-0.0293	0.0297	0.0004
	Sucrose	(Cho)OH	(DHP)O$_c$	0.0319	0.1153	-0.0278	0.0283	0.0005
		(Suc)OH$_a$	(DHP)O$_c$	0.0486	0.1539	-0.0487	0.0436	-0.0051
		(Suc)OH$_b$	(DHP)O$_d$	0.0417	0.1448	-0.0402	0.0382	-0.0020
		(DHP)OH	(Suc)O$_c$	0.0291	0.1129	-0.0249	0.0265	0.0016
	PVP	(Cho)OH	(DHP)O$_c$	0.0362	0.1252	-0.0326	0.032	-0.0006
		(DHP)OH$_a$	(PVP)O$_c$	0.0305	0.1069	-0.025	0.0259	0.0009
		(DHP)OH$_a$	(PVP)O$_d$	0.0303	0.1077	-0.0248	0.0259	0.0011
[Cho][Oac]	Glycerol	(Cho)OH	(OAc)O$_c$	0.036	0.1266	-0.0321	0.0319	-0.0002
		(Gly)OH$_a$	(OAc)O$_c$	0.0478	0.1457	-0.0463	0.0414	-0.0049
		(Gly)OH$_b$	(OAc)O$_d$	0.0432	0.1367	-0.0403	0.0372	-0.0031
	Trehalose	(Cho)OH	(OAc)O$_c$	0.0477	0.1504	-0.0472	0.0424	-0.0048
		(Tre)OH$_a$	(OAc)O$_c$	0.0219	0.0789	-0.016	0.0178	0.0018
		(Tre)OH$_b$	(Cho)O$_c$	0.0233	0.0808	-0.0167	0.0185	0.0018
		(Tre)OH$_c$	(OAc)O$_d$	0.0426	0.1324	-0.039	0.0361	-0.0029
	Trehalose-Glycerol	(Cho)OH	(Cho)O	0.0433	0.1435	-0.0416	0.0387	-0.0029
		(Tre)OH$_a$	(OAc)O$_d$	0.0432	0.1414	-0.041	0.0382	-0.0028
		(Tre)OH$_b$	(Gly)O$_c$	0.0372	0.1304	-0.0337	0.0331	-0.0006
		(Gly)OH$_a$	(Cho)O$_c$	0.0378	0.1268	-0.0336	0.0326	-0.0010
		(Gly)OH$_b$	(OAc)O$_d$	0.0402	0.1324	-0.0367	0.0349	-0.0018
	Sucrose	(Cho)OH	(OAc)O$_c$	0.0306	0.1092	-0.0254	0.0264	0.0010
		(Suc)OH$_a$	(OAc)O$_c$	0.0472	0.1480	-0.0463	0.0416	-0.0047
		(Suc)OH$_c$	(OAc)O$_d$	0.0434	0.1409	-0.0412	0.0382	-0.0030
	PVP	(Cho)OH	(PVP)O$_c$	0.0234	0.0951	-0.0186	0.0212	0.0026
